# Behçet’s Disease: An Overview of Etiopathogenesis

**DOI:** 10.3389/fimmu.2019.01067

**Published:** 2019-05-10

**Authors:** Pietro Leccese, Erkan Alpsoy

**Affiliations:** ^1^Rheumatology Institute of Lucania (IRel) and the Rheumatology Department of Lucania, San Carlo Hospital of Potenza and Madonna delle Grazie Hospital of Matera, Potenza, Italy; ^2^Department of Dermatology and Venereology, School of Medicine, Akdeniz University, Antalya, Turkey

**Keywords:** Behçet's disease, etiology, genetics, immunology, infectious agents

## Abstract

Behçet's disease (BD) is a systemic inflammatory disease with a chronic, relapsing-remitting course of unknown etiology hallmarked predominantly by mucocutaneous lesions and ocular involvement. BD shares some common features with autoimmune and autoinflammatory diseases and spondyloarthropathies (MHC-I-opathies). It is related to more than one pathogenic pathway triggered by environmental factors such as infectious agents in genetically predisposed subjects. The interplay between genetic background and immune system is linked to the BD presentation. Genetic factors have been investigated extensively, and several recent genome-wide association studies have confirmed HLA-B^*^51 to be the strongest genetic susceptibility factor. However, new non-HLA susceptibility genes have been identified. Genetic variations in the genes encoding the cytokines could affect their function and be associated with disease susceptibility. Infectious agents such as *Streptococcus sanguinis* or the differences in salivary or gut microbiome composition can be considered to trigger the innate-derived inflammation, which is, subsequently, sustained by adaptive immune responses. Altered trimming of microbial and/or endogenous peptides by endoplasmic reticulum aminopeptidase 1 (ERAP1), presented by HLA-B^*^51, may play a key role in BD pathogenesis causing an alteration in T cell balance with downregulation of Tregs and expansion of Th1 and Th17. The activity of neutrophils is increased and there is an intense neutrophil infiltration in the early stage of inflammation in organs affected by the disease. Association with HLA-B^*^51 and increased IL-17 response seems to have an important role in neutrophil activity. In this paper, we provide an overview of the most recent advances on BD etiopathogenesis.

## Introduction

Behçet's disease (BD) is a systemic inflammatory disease with a chronic, relapsing-remitting course, and its etiology is still unknown. The disease is characterized by a range of clinical manifestations including oral aphthae, genital ulcers, skin lesions, ocular, vascular, articular, gastrointestinal, urogenital, pulmonary, and neurologic involvement. BD is prevalent in regions along the “Silk Road,” extending from Japan to Mediterranean countries. BD often begins between the ages of 20–40. The disease is equally distributed between men and women and the diagnosis can be made only on the basis of clinical symptoms and signs. The course of the disease is more severe in male patients with younger age at onset and an increased number of organs affected at diagnosis (1). Disease can be recognized by clinical findings because of the absence of a universally accepted diagnostic laboratory test. BD diagnosis is largely based on mucocutaneous symptoms which are a common characteristic of various diagnostic criteria used in the diagnosis of the disease so far (2). The International Study Group for Behçet's disease criteria (requires the presence of oral ulcer plus any two of recurrent genital ulcer, typical eye lesions, typical cutaneous lesions, or a positive skin pathergy test) is the most commonly used and internationally recognized diagnostic criteria by the authors of this field (2, 3).

Besides considerable morbidity, BD has increased mortality because of the pulmonary artery and large vessel, neurological, and gastrointestinal involvements. Therefore, knowing the etiopathogenesis of BD is extremely important to better understand the disease and, more importantly, to develop targeted therapies. BD has been listed among autoimmune diseases by some authors because of positive response to classical immunosuppressive agents and involvement of autoantigens and antigen-specific T cells. Others claim the disease should be included in the group of autoinflammatory diseases because of unprovoked episodes of inflammation without evidence of antigen-specific T cells or autoantibodies, increased activity of neutrophils, elevated levels of interleukin (IL)-1β (4). Most authors evaluate the disease as a spondyloarthropathy (MHC-I-opathy) based on Human Leukocyte Antigen (HLA) class I association and epistatic endoplasmic reticulum aminopeptidase 1 (ERAP-1) interactions, increased T helper (Th) 17 type immune response, neutrophilic inflammation and barrier dysfunction in environmentally exposed organs (5). According to the current literature, BD cannot be definitely classified under any of these three groups and defining it as autoimmune, autoinflammatory or spondyloarthropathy appears to be a simplified approach (6). BD shares some common features with all the above-mentioned entities and involves more than one pathogenic pathway triggered by environmental factors such as infectious agents in genetically predisposed subjects. We will discuss the most recent evidences on the etiology of BD under the subtitles of infectious, genetic and immunological etiology sections of this review (1, 7, 8).

## Infections

Infectious agents have long been proposed as triggering factors in BD development. Antigens from viruses such as herpes simplex virus (HSV)-1 or bacteria belonging to *Streptococcus* species such as *Streptococcus sanguinis* have been suspected to have high homology with human proteins such as heat-shock proteins (HSP) and the cross-reaction leads to an immune response in genetically predisposed individuals (1, 9). Professor Hulusi Behçet was indeed one of the first authors who regarded the disease as possibly related to an infectious agent (10). Several studies have investigated the association between HSV-1 and BD. Studd et al. in an situ DNA-RNA hybridization method, detected a higher frequency of hybridization between HSV-1 DNA and complementary RNA in mononuclear cells of BD patients compared with healthy controls. The results show the presence of at least a portion of the HSV-1 genome in mononuclear cells of BD patients (11).

Several *Streptococcus* strains have become increasingly important in infectious etiology. The development of some clinical manifestations of the disease in hypersensitivity tests against streptococcal antigens is one of the most relevant evidences (12). In addition, antibodies against *S. sanguinis* and *S. pyogenes* were obtained more frequent in BD patients than in controls (13). Streptococcal 65-kDa HSP from an uncommon serotype (KTH-1, strain BD113-20) of oral *S. sanguinis* has been reported to be an important trigger in the pathogenesis (14). Neurofilament medium (Nf-M) was recently suggested as possible antigen able to trigger an immune response via molecular mimicry with bacterial HSP-65 (15). Immunoglobulin M in BD patients has been reported able to react with some streptococcal proteins such as streptococcal α-enolase and glyceraldehyde 3-phosphate dehydrogenase (16).

Cho et al. demonstrated that the *S. sanguinis* GroEL protein is a target of the serum anti-*S. sanguinis* IgA antibody. In addition, serum IgA reactivity against recombinant *S. sanguinis* GroEL has been correlated to reactivity against recombinant human hnRNP A2/B1 suggesting how autoreactive lymphocytes may be activated by infectious triggering (17).

As BD usually starts from the oral mucosa, it has been speculated that oral microbial flora may be implicated in the pathogenesis of the disease (18). BD patients can develop new-onset oral ulceration or experience both cutaneous and systemic flare-ups following dental procedures or surgical treatments for chronic tonsillitis (19, 20). Antimicrobial agents have been used successfully for treating various disease symptoms (21). Several previous studies and our experience showed oral health impairment in BD patients compared with healthy subjects (18, 22, 23). Oral health improvement in BD patients may positively modify their disease course. Dental treatments in BD patients could be associated with a relapse of oral aphthae in the short time but could decrease their number in longer follow-up (~6 months) (24), also leading to better oral health in the long-term follow-up. Higher levels of various *Streptococci* were found in the oral mucosa of BD patients. In addition, *S. sanguinis* strain resulted able to induce the secretion of inflammatory cytokines by the KTH-1 cells. It is plausible that an inflammation process induced by infectious agents in subjects with predisposing genetic background leads to the development of BD (25, 26).

No association between BD and other bacterial species such as *Borrelia burgdorferi* and or *Helicobacter pylori* have been found (27, 28). Cytomegalovirus, Epstein-Barr virus, Parvovirus B19, Varicella zoster virus, Hepatitis virus have also been investigated as possible triggering factors but these studies were characterized by low-level evidences (29, 30).

Recent studies have shown that the differences in salivary or gut microbiome composition may have a role in the pathogenesis. In a study of the salivary microbiome using high- throughput sequencing of the 16S rRNA V4 region, Coit et al. reported that BD patients have a significantly less diverse microbial community structure than healthy controls (31). In another study, Consolandi et al. compared the fecal microbiota of BD patients to healthy controls. They reported both a peculiar dysbiosis of the gut microbiota and a significant decrease of butyrate production in BD patients. Authors speculated that a defect of butyrate production might lead to both reduced T regulatory cells (Tregs) responses and activation of immunopathological T-effector responses (32).

In summary, until now, no infectious agent has been isolated as the specific etiologic agent. Additionally, results of antibacterial and antiviral treatments are controversial. However, there is a general agreement that infectious agents or microbiome is not directly responsible for the emergence of BD, but they play a triggering role in the development of the disease by causing dysfunction of the immune system.

## Genetics

Increased prevalence of the disease along the ancient “Silk Road,” familial aggregation, association with the genes inside the major histocompatibility complex (MHC) region and outside the MHC region are the main evidence of genetic influence and a complex inheritance model of disease (33, 34). The strongest genetic susceptibility factor for BD is located inside the MHC class I region including the Human Leukocyte Antigen-B51 (HLA-B^*^51). The odds ratio for individuals carrying HLA-B^*^51/B5 allele to develop BD compared with no-carriers was found to be 5.78 (33).

Genome-wide association studies (GWAS) have clearly shown the role of several single nucleotide polymorphisms (SNPs) in the etiopathogenesis of various diseases, including BD (35–41). In a multicenter study, Hughes et al. studied the association between HLA-B^*^51 and BD as well as other risk loci within the HLA region: 8572 variants were screened, and imputation and meta-analysis of 24834 variants were performed in two independent groups of BD patients. The most significant association was with rs116799036, which is located between *HLA-B* and MHC Class I Polypeptide-Related Sequence A *(MICA)* (42). Recently, Takeuchi et al. genotyped 1900 Turkish BD and 1779 controls with the *Immunochip* and demonstrated that the major BD-related polymorphism was known as rs1050502, an HLA-B^*^51 gene variant (43). However, the presence of HLA-B^*^51 alone only partially explains the genetic disease risk and all clinical manifestations of BD. Several recent GWAS have confirmed the association between BD and HLA-B^*^51, except for Fei et al's investigation. These studies also revealed new susceptibility loci both on other HLA Class I regions and on non-HLA genes (35–41). These genes provide a significant role in understanding disease pathogenesis and offer novel treatment strategies.

In general, BD-associated gene polymorphisms were localized in molecules responding to microorganisms, as well as in genes encoding cytokines and adhesion molecules. Polymorphisms within genes encoding the cytokines may affect their function and may be associated with disease predisposition (44). Researchers identified several non-HLA genetic associations by GWAS including *ERAP1, IL23 receptor (IL23R), IL-23R/IL-12RB2, IL-10*, and *STAT* genes (38, 45).

*ERAP1* variations have been identified as significant predictive loci of BD susceptibility. The gene encodes an amino-peptidase having the critical role to trim N-terminal of peptides. This mechanism was affected by the amino acids sequence of the corresponding protein (46–51). *ERAP1* is characterized by several common polymorphisms encoding variant amino acids related not only to BD, but also to ankylosing spondylitis (AS) and psoriasis (47–51). The same SNPs associated to BD risk resulted protective against AS and psoriasis: this effect depends on the different HLA interacting with *ERAP1* (46, 49). *ERAP1* polymorphisms was a risk factor preferentially in BD patients with HLA-B^*^51-positivity; *ERAP1* rs17482078 (p.Arg725Gln) might influence the peptide repertoire binding to HLA-B^*^51 (47). A recent paper suggested the critical role of the altered peptide presentation by HLA-B^*^51 in influencing disease pathogenesis (52). Based on these alterations, T-cell and natural killer (NK) cell recognition were probably affected, providing the basis for the association of *ERAP1* and HLA-B^*^51 with BD (53). A very recent alternative pathogenic hypothesis linking HLA-B^*^51 with BD involves the gut microbiome and the HLA-B^*^51 misfolding. Both ER stress and unfolded proteins were consequences of the misfolding and also the inflammation trigger. Some combination of the misfolded proteins probably influences BD pathogenesis, but this point has not yet been addressed in BD patients and several small studies reported a role in AS pathogenesis with HLA-B-27 (52).

The association between SNPs of *IL-10* and *IL-23R/IL-12RB2* genes and BD was demonstrated in Turkish (35, 40) and Japanese population (35, 39). A reduced mRNA expression in BD patients monocytes was recognized in the presence of the A-allele of rs1518111 *IL-10* compared with wild-type G-allele. PBMCs or monocytes produced significantly less IL-10 following stimulation with Toll-like receptor (TLR) ligands in individuals homozygous for A-allele of rs1518111 (35). Afkari et al. showed that *IL-10* rs1800872 A allele contributes to BD genetic risk by modulating IL-10 expression: BD patient group showed lower gene expression levels compared to the controls (54). Most disease-associated GWAS variants were found to be localized on the IL-23R side of the hotspot. These results indicate the association of BD with IL-23R rather than IL-12RB2. The association of *IL23R* rs17375018 and a haplotype of four gene variations and BD was reported, but no functional data were available for this variation. Targeted resequencing of *IL23R* in BD Japanese and Turkish patients showed novel association pieces of evidence including the reduced frequency of those rare missense variations with a protective role by reduced IL-23-dependent IL-17 production, as demonstrated in Crohn's disease (35). IL23 induces T cell activation for IL17 production and is one of the most significant activators of Th17 pathway (1). The association between BD susceptibility and *IL23R-IL12RB2* locus was confirmed in a Korean population: the intergenic rs1495965 SNP was significantly related with BD risk both in discovery and replication phases (55).

Association between STAT4 rs7574070 and BD was underlined in different studies (35, 37, 38). In addition, the disease-associated A allele was related to increased gene expression, greater severity of disease course and higher IL-17 production (35). *IL1A*-*IL1B, IRF8*, and *CEBPB-PTPN1* were three novel disease markers recently identified by direct genotyping in GWAS besides *ADO-EGR2* discovered by imputation (43).

The variation of the promoter region of *TNF* has also been reported as a risk marker for BD. Alterations of TNF expression related to gene polymorphisms may be responsible for the higher cytokine activity (56, 57). Polymorphic alleles were more frequent in BD patients and were related to higher TNF production by monocytes or mononuclear cells (45, 57). Mutations in the Mediterranean fever gene were also considered additional BD susceptibility factors (45).

The role of other genes located outside the HLA region, encoding chemokines (e.g., CCR1-CCR3, CCR5), cytokines (such as for IL-1β, IL-6, IL-8, IL-12, IL-17, IL-18, IL-23), oxidative stress-related proteins (glutathione transferase and myeloperoxidase), cell membrane receptors (TNFRSF1A, TLR2, 4, 7, 9), immunoregulatory proteins (e.g., IRF1, IRF5, CTLA-4, NF-jB), extracellular proteins (like ICAM- 1, MMP-9), and others including those for KLRC4, TNFAIP3, DEFA1, NEMO, NOD2, TLR4, and FUT2 were analyzed in several investigations with conflicting findings (45, 58–62).

Besides genetic contribution, also epigenetic processes, such as DNA methylation, histone modification, and non-coding RNAs, microRNA (miRNA) in particular, have been suggested as involved in BD pathogenesis (45, 63). The epigenetic aspects were also investigated by analyzing miRNA signatures associated with BD patients with active disease and showed that miRNAs target pathways relevant in BD, such as TNF, IFN-γ, and vascular endothelial growth factor receptor signaling cascades (64, 65). Alipour et al. reported that disease pathogenesis could be affected by altered methylation levels of interspersed repetitive sequences (IRs) elements, as well as by histone modifications and miRNA regulation, in particular, higher levels of miR-182 and miR-3591-3p and lower levels of miR-155, miR-638 and miR-4488 (63).

Recently, Zhou et al. screened a Caucasian family formed by an affected mother and two affected daughters presenting with oral and genital ulcers, uveitis, and arthralgia/arthritis clinical signs. Exome sequencing revealed two strong candidate variants, p.C78W of *TNFRSF9* and p.L227X of *TNFAIP3* genes. These mutations affect immune cell survival and proinflammatory cytokine production. Therefore, one or two of this mutation may contribute to this dominantly-inherited condition and can help us to understand how BD symptoms develop (66).

## Immunity

Activated innate immunity plays an important role in the pathogenesis of BD. Microbial triggers are sensed and processed by the innate immune system via pathogen-related and/or danger-associated molecular patterns. Overproduction of inflammatory cytokines by innate immune cells such as macrophages and dendritic cells may cause a higher production of adaptive Th1- and Th17-related cytokines. BD lesions in their early stages are predominated by neutrophils which are major immunoregulatory cell group of the innate immune system. Another member of innate immunity, natural killer (NK) cells are also found in BD lesions (67).

BD is considered as a neutrophilic vasculitis and the role of neutrophils in BD pathogenesis has long been known (7). Surface molecules, indicating neutrophil activation status (CD10, CD14, and CD16), oxidative burst and phagocytic function of neutrophils have been explored and the presence of proactive neutrophils in BD patients was reported (68). Tissue injury in BD can be modulated by neutrophils in several manners: neutrophils were hyperactivated, probably HLA B^*^51-associated, and usually were involved in perivascular infiltration (68, 69). No significant differences were observed in the oxidative burst, phagocytic microbicide activities or cytokine pattern when BD patients and controls were compared in Perazzio and colleagues' study. However, significant differences in phagocytic dysfunction were found in patients with severe active disease compared with subjects with mild disease (45, 70). In addition, the structural and functional modification of fibrinogen resulted related to reactive oxygen species and neutrophil activation via neutrophil NADPH oxidase (69). Therefore, neutrophil activation was considered as the main source of oxidative stress through the oxidation of proteins. Hyper-activated neutrophils secrete some cytokines that are both autocrine and also stimulate Th1 cells (45). Recently, Yavuz et al. reported that testosterone causes a significant neutrophil activation together with Th-1 type immune alterations which may explain a more aggressive disease with a higher mortality rate in male BD patients (71).

NK cells were also identified in BD lesions where seems they have a role in driving the CD4+ Th1 response which is the main feature of BD lesions (72, 73). However, several studies underlined increased NK cells in the peripheral blood, in particular during the active phases of the disease (72, 74, 75).

Dysregulation of the immune system contributes to BD etiopathogenesis, with increased systemic levels of inflammatory cytokines (45, 72). It's well known that CD4+T cells can differentiate into two types: Th1 cells subset, which secretes IFN-γ, IL-2, and TNF and promotes cell-mediated immunity, and Th2 cells, which produce IL-4, IL-5, IL-10, and IL-13 and promote antibody-mediated immunity (76).

The alteration of T cell balance, especially Th1/Th17 expansion and decreased regulation by Tregs, are supposed to have a significant role in BD pathogenesis (7, 43). In particular, increased frequencies of Th17 cells were reported in the BD cutaneous lesions (77). Th17 and IL-17 pathways might have a part in the development and/or activity of BD (1). Increased production of IL-17, IL-23, and IFN-γ by PBMCs besides increased frequencies of IL-17 and IFN-γ producing T cells in BD patients with active uveitis was reported (78). IL-17 levels of BD patients with active stages of uveitis, oral and genital ulcers and articular symptoms were significantly higher compared with patients with inactive stages of the same symptoms. Hamzaoui et al. demonstrated that the percentage of circulating Th17 cells and plasma interleukin IL-17 levels were increased in active BD (52, 79). Increased neutrophil activity and neutrophil infiltration in the affected organs of BD might be caused by the increased IL-17 response (80). A recent study reported that, under Th17-stimulating conditions, T cells express both IL-17 and IFN-γ. Production of large amounts of IL-17 and IFN-γ by all lymphocyte subsets in BD patients were associated with increased innate responses, early tissue neutrophil infiltrations and late adaptive immunity (67). Moreover, in experimental autoimmune uveitis (EAU) the role of Herpesvirus entry mediator (HVEM), a member of the Tumor Necrosis Factor Receptor family, has been evaluated. The HVEM seemed to be involved as a co-signaling molecule inducing both Th1 and Th17 responses in EAU. In addition, in the same mouse model, the use of anti-HVEM antibodies blocking HVEM co-signal ameliorated EAU (81).

Takeuchi et al. compared the proinflammatory and Th1-, Th2-, and Th17-related cytokines frequency in a group of BD patients with recurrent uveitis and a group of remitted uveitis before and after infliximab treatment. They found higher levels of IL-1β, IL-4, IL-17A, IL-17F, IL-21, IL-22, IL-31, IFN-γ, sCD40L, and TNF-α, with a significant difference for IL-17F, in BD-recurrent uveitis patients respect to the BD-remitted uveitis group, before drug infusion. In addition, only IL-10 levels were found higher in the remission group than in the other group (82). Emmi et al. showed that cytotoxic Th1 and Th 17 cells can play a role in inducing mucosal damage during the early stages in BD patient with active intestinal involvement (83). These results confirm that Th17 and IL-17 pathway are active and play an important role, particularly in acute attacks of the disease. Conversely, a reduction in Tregs and cytokine IL-10 were notified in the disease (72, 84).

Due to recent progress in molecular methods and basic scientific researches, our knowledge about the disease has considerably increased. GWAS have become a very important step in understanding BD pathogenesis. New genes such as ERAP1 have been introduced which help to understand the possible pathogenic mechanism of HLA-B^*^51. In the future, similar studies in different populations with a higher number of patients will provide significant advances in the etiopathology of BD. Despite all these advances, clinical expression of the disease is quite heterogeneous and show regional differences. The underlying environmental and genetic factors of this situation are not fully elucidated. Being a complex disease, BD is related more than one pathogenic pathway. Although, management of the disease has evolved noticeably because of more effective and targeted therapies we still need new treatment options for severe and non-responsive cases such as biological treatments developed for the underlying etiopathological mechanism (85).

**Figure 1:**
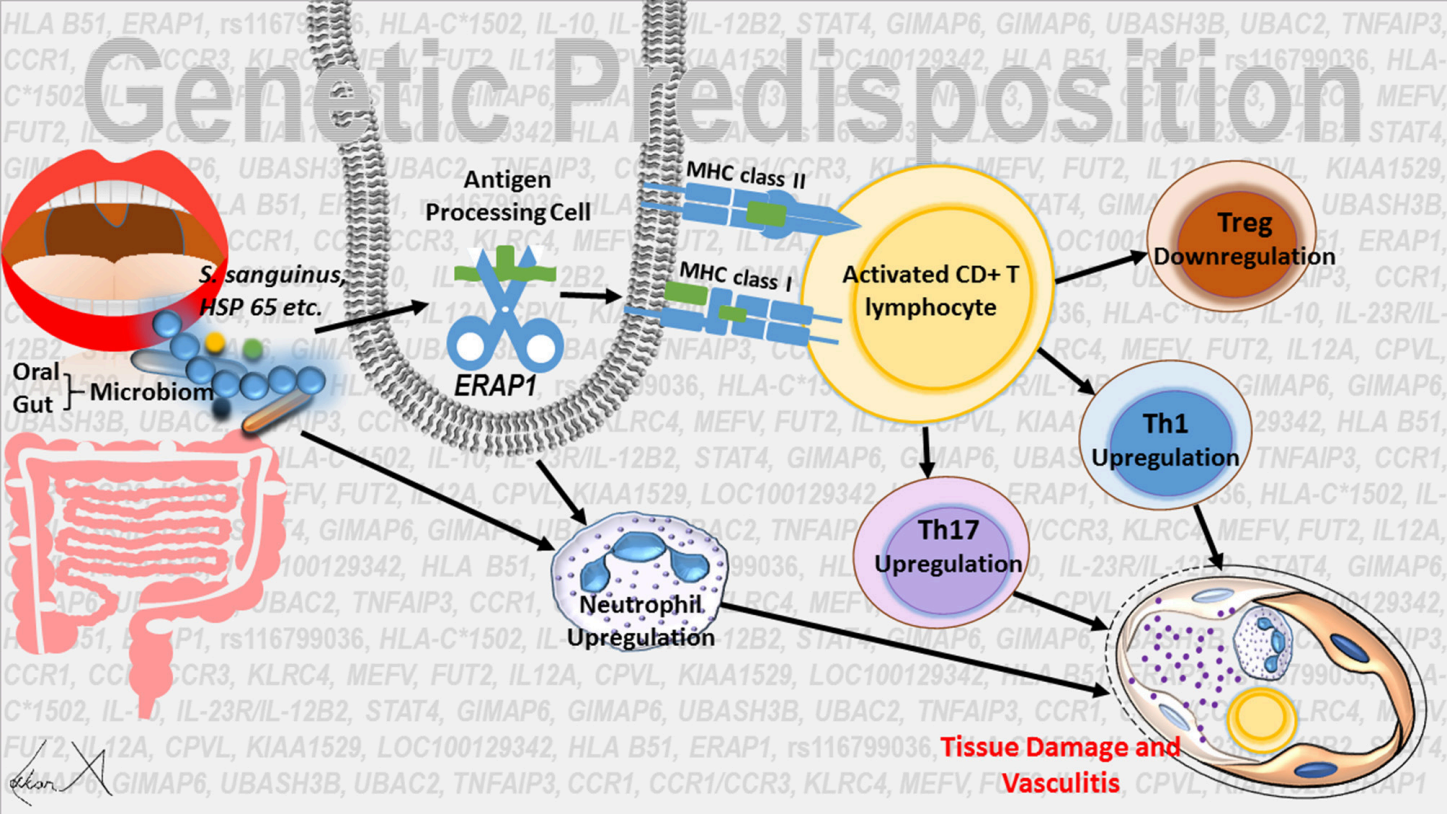
Possible regulation mechanisms in the etiopathogenesis of Behçet's disease.

In conclusion, environmental factors (*S. sanguinis* etc.) or the differences in salivary or gut microbiome composition can trigger the innate-derived inflammation, which may be subsequently sustained by adaptive immune responses. Epistatic interactions between HLA-B^*^51 and *ERAP1* variants seems to cause T cell homestasis perturbation, especially Th1 and Th 17 activation and Tregs response suppression. The activity of neutrophils is increased and there is an intense neutrophil infiltration in the early stage of inflammation in organs affected by the disease. Association with HLA-B^*^51 and increased IL-17 response have a key role in the neutrophil activity (Figure 1).

## Author Contributions

All authors listed have made a substantial, direct and intellectual contribution to the work, and approved it for publication.

### Conflict of Interest Statement

The authors declare that the research was conducted in the absence of any commercial or financial relationships that could be construed as a potential conflict of interest.
